# Hypogonadism in Women with Prader-Willi Syndrome—Clinical Recommendations Based on a Dutch Cohort Study, Review of the Literature and an International Expert Panel Discussion

**DOI:** 10.3390/jcm10245781

**Published:** 2021-12-10

**Authors:** Karlijn Pellikaan, Yassine Ben Brahim, Anna G. W. Rosenberg, Kirsten Davidse, Christine Poitou, Muriel Coupaye, Anthony P. Goldstone, Charlotte Høybye, Tania P. Markovic, Graziano Grugni, Antonino Crinò, Assumpta Caixàs, Talia Eldar-Geva, Harry J. Hirsch, Varda Gross-Tsur, Merlin G. Butler, Jennifer L. Miller, Paul-Hugo M. van der Kuy, Sjoerd A. A. van den Berg, Jenny A. Visser, Aart J. van der Lely, Laura C. G. de Graaff

**Affiliations:** 1 Department of Internal Medicine, Division of Endocrinology, Erasmus University Medical Center Rotterdam, 3015 GD Rotterdam, The Netherlands; k.pellikaan@erasmusmc.nl (K.P.); y.benbrahim@erasmusmc.nl (Y.B.B.); a.rosenberg@erasmusmc.nl (A.G.W.R.); k.davidse@erasmusmc.nl (K.D.); s.a.a.vandenberg@erasmusmc.nl (S.A.A.v.d.B.); j.visser@erasmusmc.nl (J.A.V.); a.vanderlelij@erasmusmc.nl (A.J.v.d.L.); 2 Center for Adults with Rare Genetic Syndromes, Department of Internal Medicine, Division of Endocrinology, Erasmus University Medical Center Rotterdam, 3015 GD Rotterdam, The Netherlands; 3 Dutch Center of Reference for Prader-Willi Syndrome, 3015 GD Rotterdam, The Netherlands; 4 Academic Centre for Growth Disorders, Erasmus University Medical Center Rotterdam, 3015 GD Rotterdam, The Netherlands; 5 Rare Diseases Center of Reference ‘Prader-Willi Syndrome and Obesity with Eating Disorders’ (PRADORT), Nutrition Department, Pitié-Salpêtrière Hospital, Assistance Publique-Hôpitaux de Paris, F-75013 Paris, France; christine.poitou-bernert@aphp.fr (C.P.); muriel.coupaye@aphp.fr (M.C.); 6 International Network for Research, Management & Education on adults with PWS (INfoRMEd-PWS); tony.goldstone@imperial.ac.uk (A.P.G.); charlotte.hoybye@sll.se (C.H.); tania.markovic@sydney.edu.au (T.P.M.); g.grugni@auxologico.it (G.G.); a.crino@tiscali.it (A.C.); 7 ENDO-ERN (European Reference Network); 8 PsychoNeuroEndocrinology Research Group, Centre for Neuropsychopharmacology, Division of Psychiatry, and Computational, Cognitive and Clinical Neuroimaging Laboratory, Department of Brain Sciences, Faculty of Medicine, Imperial College London, Hammersmith Hospital, London W12 0NN, UK; 9 Department of Endocrinology, Imperial College Healthcare NHS Trust, Hammersmith Hospital, London W12 0HS, UK; 10 Department of Molecular Medicine and Surgery, Karolinska Institutet, 17176 Stockholm, Sweden; 11 Center for Adults with Rare Genetic Syndromes, Department of Internal Medicine, Division of Department of Endocrinology, Karolinska University Hospital, 17176 Stockholm, Sweden; 12 Metabolism & Obesity Services, Royal Prince Alfred Hospital, Camperdown, NSW 2050, Australia; 13 Charles Perkins Centre, Faculty of Medicine and Health, University of Sydney, Sydney, NSW 2006, Australia; 14 Divison of Auxology, Istituto Auxologico Italiano, IRCCS, 28824 Piancavallo, Italy; 15 Reference Center for Prader-Willi Syndrome, Bambino Gesù Hospital, Research Institute, 00050 Palidoro, Italy; 16 Endocrinology and Nutrition Department, Institut d’Investigació I Innovació Parc Taulí I3PT, Parc Taulí Hospital Universitari, Department of Medicine, Universitat Autònoma de Barcelona, 08208 Sabadell, Spain; 17 The Israel Multidisciplinary Prader-Willi Syndrome Clinic, Jerusalem 9103102, Israel; gevat@szmc.org.il (T.E.-G.); hirschmd@gmail.com (H.J.H.); varda.gross@gmail.com (V.G.-T.); 18 Reproductive Endocrinology and Genetics Unit, Department of Obstetrics and Gynecology, Shaare-Zedek Medical Center, Jerusalem 9103102, Israel; 19 Hebrew University School of Medicine, Jerusalem 9112102, Israel; 20 Department of Pediatrics, Shaare Zedek Medical Center, Jerusalem 9103102, Israel; 21 Neuropediatrics Unit, Department of Pediatrics, Shaare Zedek Medical Center, Jerusalem 9103102, Israel; 22 Departments of Psychiatry, Behavioral Sciences and Pediatrics, University of Kansas Medical Center, Kansas City, KS 66160, USA; mbutler4@kumc.edu; 23 Department of Pediatrics, College of Medicine, University of Florida, Gainesville, FL 32610, USA; millejl@peds.ufl.edu; 24 Department of Hospital Pharmacy, Erasmus University Medical Center Rotterdam, 3015 GD Rotterdam, The Netherlands; h.vanderkuy@erasmusmc.nl; 25 Department of Clinical Chemistry, Erasmus University Medical Center Rotterdam, 3015 GD Rotterdam, The Netherlands

**Keywords:** Prader-Willi syndrome, hypogonadism, hypothalamus, pituitary gland, estrogens, menstrual cycle, obesity, puberty

## Abstract

Prader-Willi syndrome (PWS) is a rare neuroendocrine genetic syndrome. Characteristics of PWS include hyperphagia, hypotonia, and intellectual disability. Pituitary hormone deficiencies, caused by hypothalamic dysfunction, are common and hypogonadism is the most prevalent. Untreated hypogonadism can cause osteoporosis, which is already an important issue in PWS. Therefore, timely detection and treatment of hypogonadism is crucial. To increase understanding and prevent undertreatment, we (1) performed a cohort study in the Dutch PWS population, (2) thoroughly reviewed the literature on female hypogonadism in PWS and (3) provide clinical recommendations on behalf of an international expert panel. For the cohort study, we retrospectively collected results of a systematic health screening in 64 female adults with PWS, which included a medical questionnaire, medical file search, medical interview, physical examination and biochemical measurements. Our data show that hypogonadism is frequent in females with PWS (94%), but is often undiagnosed and untreated. This could be related to unfamiliarity with the syndrome, fear of behavioral changes, hygienic concerns, or drug interactions. To prevent underdiagnosis and undertreatment, we provide practical recommendations for the screening and treatment of hypogonadism in females with PWS.

## 1. Introduction

Prader-Willi syndrome (PWS) is a rare, complex, neuroendocrine syndrome with an estimated prevalence of 1:10,000–1:30,000 [1]. PWS is an imprinting disorder caused by the lack of expression of a cluster of paternally expressed genes on chromosome 15q11-13. The most common genetic abnormalities are: a paternal deletion (60–75%), a maternal uniparental disomy 15 (mUPD, 20–35%), an imprinting center defect (ICD, 1–4%) or a paternal chromosomal translocation (0.1%) [2,3]. Features of adults with PWS include hypotonia, mild to moderate intellectual disability, dysmorphic features and hypothalamic dysfunction, leading to hyperphagia and early-childhood onset obesity (if uncontrolled), sleep disorders, abnormal temperature regulation, high pain threshold, and pituitary hormone deficiencies (PHD) [1,4,5,6,7]. Additionally, patients with PWS may display challenging behavior, and the prevalence of psychosis is increased, especially in patients with mUPD [8,9,10,11].

The most prevalent PHD in PWS is hypogonadism. We previously reported on hypogonadism in men with PWS [12], in this study we focused on hypogonadism in women with PWS.

The reported prevalence of hypogonadism in female adults with PWS varies between 54 and 100% [13,14,15,16,17,18,19,20,21,22]. Hypogonadism in patients with PWS is believed to be caused by hypothalamic dysfunction, but more recent studies indicate that primary ovarian dysfunction also occurs [17,19,23,24].

During infancy, females often present with hypoplasia of the clitoris and labia minora [25]. Although there is generally a normal onset of puberty, i.e., breast development, its progression is often delayed and incomplete and spontaneous menarche usually does not occur [21,23,26]. If menarche occurs spontaneously, it is often delayed (at a mean age of 20 years) and followed by irregular menstruations or secondary amenorrhea [18,24]. Despite absent menstruation and impaired maturation of follicles in many female patients with PWS [26], pregnancy has been described, although rarely [27,28,29,30,31,32].

In the general population, female hypogonadism may result in a decreased quality of life, decreased muscle strength, and osteoporosis [33,34,35,36]. Additionally, there are indications that estradiol has beneficial effects on the cardiovascular system [37,38]. Besides hypogonadism, patients with PWS also have a high prevalence of other risk factors for osteoporosis like growth hormone (GH) deficiency and low physical activity [4,39,40,41,42,43]. This makes early detection and treatment of hypogonadism especially important to avoid osteoporosis [44,45].

Based on a Dutch cohort, we report the prevalence and characteristics of hypogonadism and its treatment in female adults with PWS. Additionally, we provide an overview of the current literature on the prevalence of hypogonadism and related laboratory values in women with PWS. As there are currently no PWS-specific guidelines [46], we present a practical algorithm for the screening and treatment of hypogonadism in female adults with PWS based on an international expert panel discussion.

## 2. Materials and Methods

Ethical review and approval were waived for this study by the Medical Ethics Committee of the Erasmus University Medical Center, Rotterdam, The Netherlands (MEC-2018-1389).

We retrospectively reviewed the medical files of female adults with PWS who underwent the systematic health screening at our PWS reference center (Erasmus University Medical Center, Rotterdam, the Netherlands), between January 2015 and December 2020. As previously described (see [47]), this systematic screening consisted of a medical questionnaire, a review of the medical files, a structured interview, a complete physical examination, biochemical measurements and, if indicated and feasible, additional tests.

The results of our study about hypogonadism in male adults with PWS are reported separately [12], since the characteristics of hypogonadism and its treatment differ substantially between males and females.

### 2.1. Terminology

We will use the term hormone-replacement therapy (HRT) for tibolone and estrogen-containing preparations that cannot be used as contraception. We will use the term hypogonadism hormone therapy (HHT) as an overarching term for HRT and estrogen-containing contraceptives.

### 2.2. Definition of Hypogonadism

In obese women, estradiol levels can be within the reference range despite dysfunction of the hypothalamus, pituitary or ovaries, due to increased aromatase activity in adipose tissue [48,49]. Therefore, hypogonadism in women with PWS was defined as absent or irregular menstruation, regardless of serum estradiol levels. When females used HHT or progesterone-only contraceptives, including intrauterine devices (IUDs), before screening, we asked about the menstrual cycle before treatment or searched the medical files for information on the menstrual cycle before treatment or a previous diagnosis of hypogonadism. When females were over 50 years old, we asked about the menstrual cycle before they reached menopausal age. Central hypogonadism was defined as absent or irregular menstrual cycle either (1) in the presence of serum luteinizing hormone (LH) and follicle-stimulating hormone (FSH) concentrations below the reference range, or (2) combined with a low estradiol concentration in the presence of serum LH and FSH concentrations within the reference range. Primary hypogonadism was defined as an absent or irregular menstrual cycle with serum LH and FSH concentrations above the reference range. Due to intellectual disability in most patients, gynecological evaluation was not performed routinely.

### 2.3. Laboratory Measurements

As part of regular hospital visits, blood samples were collected for general medical screening, including measurement of LH, FSH, estradiol and sex hormone binding globulin (SHBG). When females were treated with HHT or progesterone-only contraceptives (including IUDs as these may have systemic effects [50]), only LH, FSH and estradiol values from before the start of these preparations were included. If no SHBG value from before the start of these preparations was available, an SHBG value during treatment was used. Anti-Müllerian hormone (AMH) levels were not routinely measured as this would not have any consequences for treatment in this patient population.

Estradiol concentrations were measured using the Roche Elecsys assay (reference range 55–1285 pmol/L). LH and FSH concentrations were measured using the Siemens Immulite 2000XPi (Siemens Healthcare Diagnostics Inc., Tarrytown, NY, USA) (reference range 1.5–8.0 IU/L for LH and 2.0–7.0 IU/L for FSH) until February 2019. After that date, the Fujirebio Lumipulse G1200 (Fujirebio, Inc., Tokyo, Japan) was used (reference range 1.0–5.5 IU/L for LH and 0.8–5.1 IU/L for FSH). SHBG concentrations were measured using the Siemens Immulite 2000XPi (reference range 10–70 nmol/L) until June 2020. After that date, the IDS-ISYS (Immunodiagnostic Systems, Boldon, United Kingdom) was used (reference range 10–70 nmol/L) with a similar calibration, as confirmed by external quality assessment schemes.

### 2.4. Expert Panel Discussion on Diagnosis and Treatment of Hypogonadism

Eleven experts with experience with the treatment of hypogonadism in females with PWS (C.P., M.C., A.P.G., C.H., T.P.M., G.G., An.C., As.C., H.J.H., J.L.M. and L.C.G.d.G.) shared their experience regarding the diagnosis and treatment of hypogonadism in females with PWS and agreed to the clinical recommendations. They were also specifically asked what criteria they used to decide when treatment for hypogonadism shoud be started and at what age they think that this treatment should be discontinued.

### 2.5. Literature Search

On 24 September 2020, we performed a literature search regarding the prevalence of hypogonadism and related laboratory measurements in adults with PWS. The search was last updated on 3 June 2021. The search strategy is provided in the Supplementary Materials in Table S1. We excluded manuscripts that included less than ten adults (females and males) with PWS, manuscripts that were not available in English, and conference abstracts. When the prevalence of hypogonadism or laboratory values were not available for adults only, we asked the authors to provide this information for the adults separately. When manuscripts reported on overlapping populations, the manuscript with most patients or, when the number of patients was similar, the most recent manuscript was included. This search strategy identified manuscripts on hypogonadism in both men and women with PWS, however manuscripts that did not include women were excluded in the current literature review. The results of our literature review on hypogonadism in male adults with PWS are reported separately [12].

### 2.6. Data Analysis

We used R version 3.6.3 (R Foundation for Statistical Computing, Vienna, Austria) for all statistical analyses. For dichotomous variables we display the number and the percentage of people, *n* (%), and continuous variables are shown as median (interquartile range (IQR)). We used a Wilcoxon rank sum test to compare estradiol or SHBG concentrations between patients with an mUPD or a paternal deletion and between patients who used or did not use recombinant human growth hormone (rhGH) treatment. We used the Kendall rank correlation test to investigate the relationship between estradiol or SHBG concentrations and age or body mass index (BMI). We used linear regression models with likelihood ratio tests to correct for age. For all analyses involving FSH and LH, a variable indicating whether the measurement was performed before or after 1 February 2019 (when the method was changed with a different calibration) was included in a linear regression model. Samples with concentrations below the assay detection limit were assigned the value of the detection limit. If there were too many ties, an exact calculation method was used. No correction for multiple testing was performed in this exploratory analysis. *p*-values below 0.05 were considered statistically significant.

## 3. Results

### 3.1. Baseline Characteristics

The baseline characteristics of the Dutch adult female PWS cohort are shown in Table 1. We included 64 women with PWS. The median age was 28 years (IQR 22–37, range 18–58 years). The median BMI was 32 kg/m^2^ (IQR 27–40). The most frequent genetic subtype was paternal deletion of chromosome 15q11.2-13 (*n* = 37, 58%). Twenty-four females (38%) received rhGH treatment and 24 females (38%) used psychotropic medication at the time of the study. Frequently used types of psychotropic medication included risperidone (*n* = 9), benzodiazepines (*n* = 6), valproic acid (*n* = 4) and non-tricyclic antidepressants (*n* = 5). Six females were in a relationship with sexual intercourse, of whom three used oral contraceptives, one used progestogen-containing contraceptive injections, one used barrier contraceptives and one was sterilized.

### 3.2. Hypogonadism

Of the 64 women included in this study, information about gonadal function was available for 50 patients. Of these 50 women, 47 (94%) had hypogonadism (Table 2), of whom 30 had been diagnosed before (but 7 of these were untreated at the first visit to our outpatient clinic) and 17 were detected by our screening. Of these 50 women, two were above 50 years old (both 52 years old), but both reported that they never had had a menstrual cycle before reaching menopausal age. The median age at the start of HHT was 20 years (IQR 16–28).

Four of the 14 women in whom information about gonadal function was unavailable were already aged over 50 years and there was no information about the menstrual cycle before they had reached menopausal age. For the remaining 10 patients, neither patients nor caregivers remembered whether there had been a regular menstrual cycle before the start of estrogen- and/or progestogen-containing preparations.

Supplementary Materials Tables S2 and S3 show serum estradiol, LH, FSH and SHBG concentrations according to genetic subtype, rhGH treatment, age and BMI (no significant relationships).

**Figure 1:**
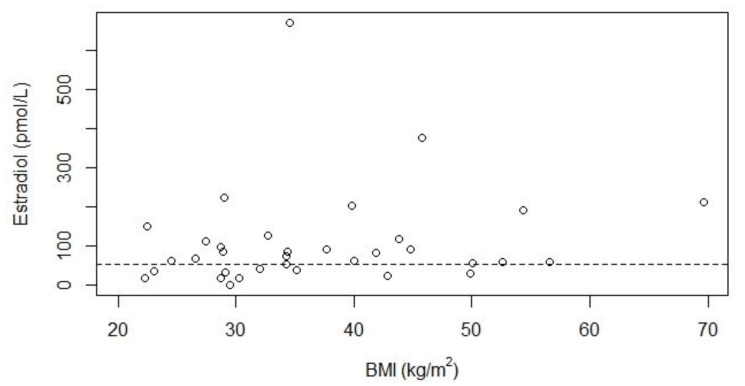
Relationship between serum estradiol concentrations and BMI for 34 women with Prader-Willi syndrome. Abbreviations: body mass index (BMI). The dotted line represents the lower limit of normal for estradiol of 55 pmol/L. Thirty-three women included in this figure had hypogonadism. One woman did not have hypogonadism as she had a regular menstrual cycle, she had an estradiol value of 41 pmol/L. The *p*-value for the relationship between estradiol and BMI was 0.13, Kendall’s Tau was 0.18.

**Figure 2:**
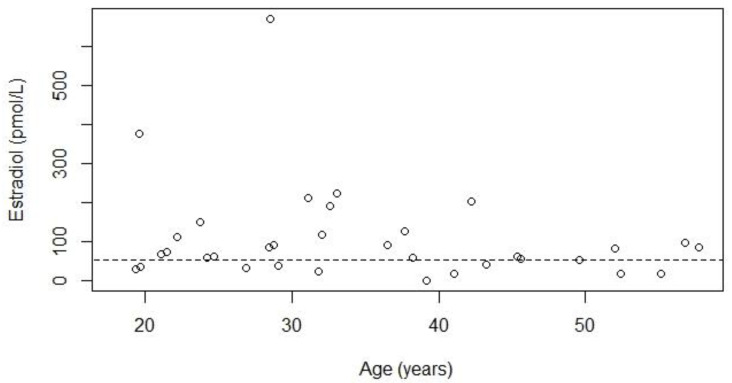
Relationship between serum estradiol concentrations and age for 34 women with Prader-Willi syndrome. The dotted line represents the lower limit of normal for estradiol of 55 pmol/L. Thirty-three women included in this figure had hypogonadism. One woman did not have hypogonadism as she had a regular menstrual cycle, she had an estradiol value of 41 pmol/L. The *p*-value for the relationship between estradiol and age was 0.27, Kendall’s Tau was −0.13.

Figure 1 and Figure 2 show estradiol levels according to BMI and age, both did not show a significant relationship. Three patients (aged 30–40 years) had spontaneous menstruation after significant weight loss, and one of these patients developed a regular menstrual cycle. These three women were still overweight after weight loss. All seven women with a BMI below 25 kg/m^2^ had hypogonadism, indicating that obesity is not the only cause of hypogonadism.

### 3.3. Types of Hypogonadism

LH, FSH and estradiol values were available for 27 women with hypogonadism (Table 3). Seven patients (26%) had central hypogonadism and one (4%) had primary hypogonadism. For the other 19 patients (70%) the type of hypogonadism could not be classified as either central or primary due to discrepant LH and FSH values. Supplementary Materials Figure S1 shows the LH, estradiol and FSH values of the 27 individual patients.

### 3.4. Hormone Treatment

At the time of the study, 47 females used estrogen- and/or progestogen-containing preparations. Twenty-six women used oral contraceptives, 18 HRT (two of whom also used progestogen-containing contraceptive injections) and one a transdermal contraceptive patch. In addition, two women without hypogonadism used progesterone-only contraception (injections and IUD).

For oral contraceptives, spontaneously reported adverse effects were spotting (*n* = 3), increase in challenging behavior with psychotic symptoms (*n* = 2), hygienic difficulties (*n* = 2), headache (*n* = 2), stomach ache (*n* = 2), weight gain (*n* = 1), and hair loss (*n* = 1).

For HRT, spontaneously reported adverse effects were spotting (*n* = 2) and increase in challenging behavior (*n* = 2).

### 3.5. Expert Panel Discussion on Diagnosis and Treatment of Hypogonadism

**Figure 3:**
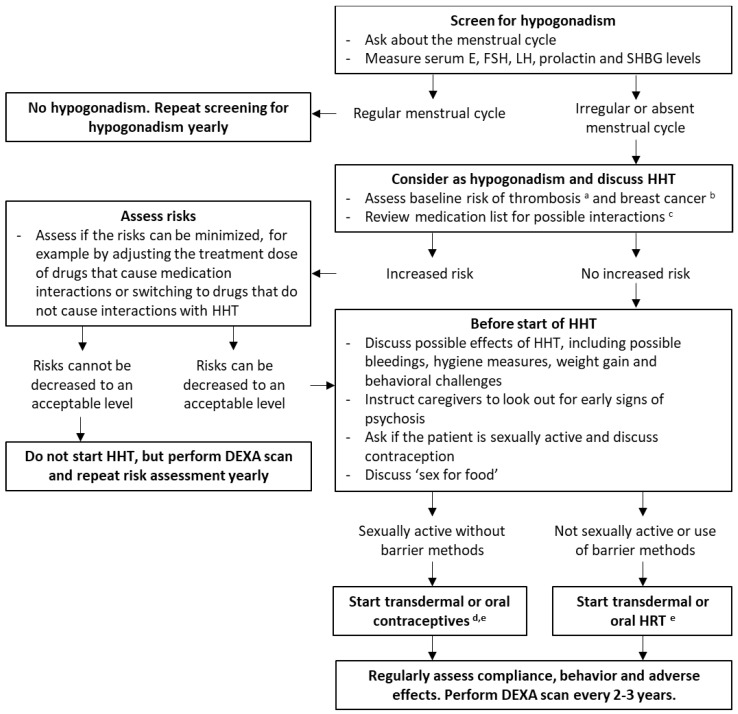
Recommendations for the treatment of hypogonadism in females with Prader-Willi syndrome, based on the results of this cohort study, a review of the literature and the expert opinion of an international panel of PWS-experts. Abbreviations: dual-energy X-ray absorptiometry (DEXA), estradiol (E), follicle stimulating hormone (FSH), hypogonadism hormone therapy (HHT), hormone replacement therapy (HRT), luteinizing hormone (LH), sex hormone binding globulin (SHBG). HRT includes tibolone and estrogen-containing preparations that cannot be used as contraception. HHT includes HRT and estrogen-containing contraceptives. **^a^** Relevant risk factors for thrombosis include: previous thrombotic events, increased age, being overweight or obese, smoking, and immobility [51]. **^b^** Relevant risk factors for breast-cancer include: breast or ovarian cancer in first degree relatives before the age of 50, genetic mutations (e.g., BRCA), being overweight or obese and alcohol abuse. **^c^** For example interactions with psychotropic medication and recombinant human growth hormone treatment. **^d^** Start the combined oral contraceptive pill or transdermal patches containing both estrogen and progestogen. An IUD combined with oral or topical estrogens may be considered. However, general anesthesia or sedation may be necessary as insertion can be traumatic in patients with an intellectual disability and (past) sexual abuse should be excluded first. **^e^** Transdermal administration is preferred due to the lower risk of thrombosis, however oral preparations could be preferred in patients with skin picking.

Eleven experts with experience with the diagnosis and treatment of hypogonadism in females with PWS agreed to the recommendations presented in Figure 3.

#### 3.5.1. Start Treatment for Hypogonadism

Nine experts in our expert panel discussion indicated that the decision to start HHT was not based on estradiol concentrations, but on the menstrual cycle (*n* = 9), bone mineral density (BMD) (*n* = 9), the ability of the patient to deal with possible vaginal bleeding (*n* = 6), sexual activity (*n* = 1) and/or desire of the patient to have ‘normal’ monthly vaginal bleedings (*n* = 1). One expert based this decision on the menstrual cycle and BMD, but also considered estradiol levels, with a cut-off of 150 pmol/L. This expert did not consider the ability of the patient to deal with vaginal bleeding an issue, as she used continuous preparations that minimised vaginal bleeding in these patients. Lastly, one expert relied only on estradiol levels (below 150 pmol/L) to start HHT.

#### 3.5.2. Stop Treatment for Hypogonadism

Two experts indicated that they did not have experience with HHT in women above 50 years old due to the decreased life expectancy of patients with PWS. One expert indicated that he did not have enough experience with this issue to formulate clinical recommendations. The other ten experts recommended to continue HHT until the natural age of menopause (around 50 years), similar to the clinical practice for non-PWS women. Five experts indicated that treatment could be continued longer (up until the age of 55–60) or be restarted in case of symptoms or low BMD.

### 3.6. Literature Review

We found ten articles that described hypogonadism or relevant laboratory values in women with PWS (Table 4 and Table 5). Seven articles used characteristics of the menstrual cycle (i.e., absent or irregular menstrual cycle) to define hypogonadism. Seven articles reported FSH, LH and estradiol values, three SHBG values and two inhibin B and AMH values. The prevalence of hypogonadism ranged from 54% to 100%, with most articles (8 out of 10) reporting a prevalence above 80%. Hypogonadism was most often central in origin (21 reported cases), although primary hypogonadism (3 reported cases) was also reported. One article reported that most females had a combined form of primary and central hypogonadism (15 reported cases).

## 4. Discussion

Hypogonadism was present in nearly all females with PWS in our cohort (94%) and was often undiagnosed (34%) and untreated. The high prevalence of hypogonadism in our cohort was in accordance with the literature.

### 4.1. Type of Hypogonadism

In our cohort, both central and primary hypogonadism were found, but in many patients the type of hypogonadism could not be determined due to discrepant LH and FSH values. The discrepancy between LH and FSH levels suggests that hypogonadism in females with PWS is not solely caused by hypothalamic dysfunction, but possibly also by combined ovarian and hypothalamic hypogonadism, as is seen in males with PWS [12,52,53]. Previous research suggests that AMH is generally normal in girls and women with PWS [21,26], which argues against primary ovarian dysfunction. However, one study showed that AMH levels in girls with PWS were significantly lower compared to controls, while a similar assay was used in all three studies [24]. Inhibin B levels are usually low or low-normal in women with PWS [21,24,26], suggesting decreased antral follicles.

### 4.2. Ovarian Histology

Literature on ovarian histology in females with PWS is scant. We found only two case reports [23]: (i) a 32 year-old woman with PWS with delayed menarche (20 years) and an irregular menstrual cycle, who became pregnant, and in whom an ovarian biopsy (performed during caesarean section) showed normal follicles in all stages of development [29]; and, (ii) a female with PWS who reported having menarche aged 11 years and a regular menstrual cycle but who died unexpectedly (age 22 years) and in whom, at autopsy, small, immature ovaries were found that showed no evidence of corpora lutea or significant follicular development [54]. In order to explain the occurrence of primary ovarian dysfunction in women with PWS, more research on the ovarian histology of these females is urgently needed.

### 4.3. Factors Influencing Hypogonadism in PWS

We did not find any significant association between laboratory measurements and genetic subtype, rhGH treatment, BMI, or age. The lack of a clear association between estradiol levels and BMI in our study might be explained by BMI’s poor reflection of adiposity in PWS, due to the abnormal body composition (low muscle mass and high fat mass) [55]. Many females with absent or irregular menses had estradiol levels within the normal range, possibly due to increased aromatase activity in adipose tissue, leading to normal serum estradiol levels despite hypothalamic, pituitary or ovarian dysfunction [48,49]. Supporting this hypothesis, three patients with amenorrhea developed a menstrual cycle after weight loss. The presence of hypogonadism in several females with a normal BMI also suggests obesity is not the only cause of central hypogonadism in females with PWS.

### 4.4. Importance of Treatment of Hypogonadism

Many beneficial effects of HHT for the treatment of hypogonadism have been described, including beneficial effects on bone health, muscle and fat, psychological outcomes and cardiovascular health. Some of these beneficial effects are especially important for patients with PWS as they already have a higher risk to develop an abnormal body composition, psychological problems and cardiovascular disease.

#### 4.4.1. Bone Health

A reduced BMD is commonly seen in patients with PWS [44,45]. Since estrogens play an important role in bone health [56,57], treating hypogonadism is important to optimize bone health. In the general population, low serum estradiol levels are associated with an increased risk of osteoporosis and osteoporotic fractures [58,59]. Both HRT and the combined oral contraceptive pill have beneficial effects on BMD [57,60,61,62]. In women with PWS, hypogonadism is also associated with a decreased BMD [63,64], for which for HRT is beneficial [64].

#### 4.4.2. Muscle and Fat

Estrogen has important effects on muscle function [35]. In women with hypopituitarism who are treated with rhGH, co-treatment with estrogen leads to an increase in lean body mass and a decrease in fat mass [65]. As patients with PWS have an abnormal body composition with a high fat mass and low lean body mass, estrogen might help to improve body composition [55].

#### 4.4.3. Psychological Effects

Untreated female hypogonadism can have negative consequences on mood, quality of life and energy level [33,34] and HHT has beneficial psychological effects in perimenopausal women [66,67,68]. However, this has not been specifically investigated in women with PWS.

#### 4.4.4. Cardiovascular Risk

Some studies in the general female population suggest that estradiol has beneficial effects on the cardiovascular system [37,38]. In women with premature ovarian insufficiency, HRT seems to have beneficial effects on endothelial dysfunction, ischemic heart disease and cardiovascular mortality [69].

### 4.5. Recommendations

Based on the potential beneficial effects, we recommend treatment of hypogonadism in all adult females with PWS. For the treatment of hypogonadism in children with PWS, we refer to the recommendations for children with PWS [23].

Patients with PWS are preferably treated by a multidisciplinary team or in a PWS reference center, experienced in treating hypogonadism in females with PWS. However, as a PWS reference center is not always available, we provide clinical recommendations for the screening and treatment of hypogonadism in women with PWS (Figure 3). We aim to highlight topics that are especially important or difficult in females with PWS. We recommend to follow non-PWS specific hypogonadism guidelines for topics not addressed here.

#### 4.5.1. Assessment of Gonadal Function

To avoid the negative consequences of long-term untreated hypogonadism, systematic screening for hypogonadism is warranted in all women with PWS. We showed that many women with PWS had estradiol values within the reference range. However, most experts in our expert panel discussion indicated that the decision to start treatment was based on the menstrual cycle and BMD rather than on serum estradiol concentrations. Therefore we recommend asking about the menstrual cycle every year and to start HHT when the menstrual cycle is absent or irregular. Additionally, a dual-energy X-ray absorptiometry (DEXA) scan should be performed as the presence of osteoporosis or osteopenia makes treatment with HHT even more urgent. Laboratory measurements could be performed to determine the type of hypogonadism and to exclude hyperprolactinemia.

#### 4.5.2. Treatment Regimen

To prevent the increased risk of endometrial cancer caused by estrogen-only preparations, a progestogen must also be prescribed [70]. The treatment regimen should be adjusted in case of sexual activity, as females with PWS are not always infertile and HRT does not protect against pregnancy. It is important to note that intellectual disability does not rule out the wish for sexual intercourse [71]. In our cohort, six females were in a relationship with sexual intercourse. As pregnancies have been described in females with PWS [27,28,29,30,31,32], it is important to discuss contraception in patients who are sexually active. It should be noted that some types of contraception, such as progestogen-only preparations, do not aid in the treatment of hypogonadism and prevention of osteoporosis and may even lead to a decrease in BMD [72,73,74].

Estrogen can be administered both orally and transdermally and with same dosage as used for non-PWS women. In case of problematic skin picking, topical gels could be preferred over transdermal patches.

#### 4.5.3. Drug Interactions

Frequently used medication in adults with PWS include psychotropic medication and rhGH treatment, both of which may interact with HHT.

##### Psychotropic Medication

Due to the increased prevalence of challenging behavior and psychosis, use of psychotropic medication is common in patients with PWS [8,9,10,11,75]. There are few clinically significant interactions between HHT and psychotropic medication. However, some theoretical interactions have been proposed [76]. HHT might influence the plasma concentrations of psychotropic medication. Both endogenous and exogenous estrogens induce the enzyme uridine 5’-diphospho-glucuronosyltransferase (UGT) 1A4, which is involved in the metabolism of some psychotropic medication [77,78,79]. Therefore, HHT might lead to increased metabolism of, for example, lamotrigine [80,81], valproic acid [81], clozapine [82], olanzapine [83,84], and amitriptyline [85], for which dose adjustment might be indicated.

Moreover, estrogens [86] and progestogens [87] are metabolized by cytochrome P450 (CYP) 1A2, 2C19 and 3A4 enzymes, which could lead to competition with psychotropic medication that are metabolized via the same enzymes. This could, for example, lead to increased clozapine concentrations [77].

Some psychotropic medication may also influence the plasma concentrations of HHT. For example, lamotrigine and carbamazepine induce the production of SHBG, which could lower serum progesterone levels and decrease the effect of hormonal contraceptives [88].

Hyperprolactinemia is a common adverse effect of some antipsychotics and hyperprolactinemia might result in hypogonadism. This could be an issue in women with mild hypogonadism, and measurements of prolactin levels in women treated with antipsychotics is recommended [89]. It could be considered to check the prolactin level before starting the antipsychotic therapy to avoid later unnecessary diagnostic investigations in case of mild hyperprolactinaemia during the psychiatric treatment.

##### Recombinant Human Growth Hormone Treatment

There is a complex interaction between GH and the gonadal system. Oral administration of estrogens inhibits hepatic insulin-like growth factor-1 (IGF-1) production, resulting in lower serum IGF-1 concentrations [65,90], while transdermal administration of estrogens may increase IGF-1 concentrations [91]. Therefore, it is important to measure serum IGF-1 concentrations 3–6 months after the start or a change in dosage of HHT and to reevaluate the dose of rhGH treatment. Transdermal HHT might enable lower doses of rhGH to be administered which may have a cost benefit.

#### 4.5.4. Intellectual Disability and Menstrual Hygiene

Monthly bleedings can cause practical problems for females with PWS and their caregivers [92], especially in patients with intellectual disability. Therefore, both patients and caregivers need to be informed about spotting or monthly bleedings, in order to take adequate hygiene measures. If monthly bleedings are deemed undesirable, preparations which do not lead to bleedings (tibolone, oral or transdermal preparations with continuous estrogen and progestogen administration or continuous estrogen tablets/patches in combination with progestogen-containing contraceptive injections) should be considered. However, patients and their caregivers should be warned that bleedings might occur during the first couple of weeks after starting these treatments. In patients who are sexually active, an IUD combined with oral or topical estrogens could also be considered. However, gynecological examination required to insert an IUD is often painful and stressful for women with intellectual disabilities, especially when they have a history of sexual abuse [93]. Therefore, general anesthesia or sedation may be necessary during insertion of the IUD [94,95] and a possible history of sexual abuse should be excluded first (see also Section 4.5.9 Sex for Food).

#### 4.5.5. Challenging Behavior and Psychotic Symptoms

In our cohort, the caregivers of four females spontaneously reported an increase in challenging behaviors after start of HHT. Two of these patients even displayed psychotic symptoms. Although the behavioral challenges seemed associated with the use of HHT, it is possible that this was a coincidence as challenging behavior and psychotic symptoms are frequent in patients with PWS. Additionally, behavioral challenges were not systematically assessed and therefore it is possible that these challenges occur even more often than reported in this study. More research is needed to assess the relationship between HHT and behavior and psychotic symptoms in females with PWS. The relation between behavioral changes and HHT can be multifactorial. One possible explanation is menstrual pain or discomfort, which can cause challenging behaviors in females with an intellectual disability [96]. However, the high pain threshold in most patients with PWS makes this explanation less likely. Another possible explanation is the visual exposure to menstrual blood, which might cause feelings of fear, especially in patients with significant intellectual disabilities and the developmental age of a young child. Based on our experience, we recommend parents and caregivers are educated about early signs of psychosis and instructed to alert a physician if these symptoms occur.

#### 4.5.6. Thrombosis

HHT can increase the risk of thrombosis [97]. As patients with PWS already have a higher risk of thrombotic events [98], it is important to avoid any other risk factors for thrombosis, if possible. Due to lower levels of sex hormones, HRT is less thrombogenic than hormone contraception [51,99,100]. Therefore, we advise to limit the use of hormone contraceptives to patients who are sexually active and unable to use non-hormonal contraception. Additionally, transdermal estrogen administration is preferred in women with PWS due to its lower risk of thrombosis compared to oral administration [101]. However, it should be noted that contraceptive patches might be less reliable in obese women [102], although results are inconclusive [103]. Lastly, it is important to address other risk factors for thrombosis including smoking, obesity and immobility.

#### 4.5.7. Weight Gain

In our cohort, one patient spontaneously reported weight gain after starting estrogen- and progestogen-containing oral contraception. Although this might have been the result of fluid retention due to oral contraceptives [104], excessive food intake should always be considered when patients with PWS gain weight.

#### 4.5.8. Breast Cancer

Two cohort studies reported an increased risk of cancer in patients with PWS [105,106], especially myeloid leukemia [106]. There are no indications that the risk of breast cancer is increased in patients with PWS. However, as in non-PWS women, it is important to consider the additional risk of breast cancer resulting from HHT and to recommend participation in national breast cancer screening programs [70,107,108].

#### 4.5.9. Sex for Food

Due to the combination of intellectual disabilities and hyperphagia, women with PWS are especially vulnerable to become victims of sexual abuse. Hyperphagia often leads to food-seeking behavior [109]. This, especially when combined with cognitive impairment, makes them easy victims of ‘sex for food’ (receiving food in exchange for sexual acts) [110]. Most of the patients do not disclose or understand the sexual abuse, resulting in continuation of the abuse [110]. We recommend that this important topic is addressed in all women with PWS, in order to avoid negative psychological consequences and possible pregnancies. Discussing hypogonadism and HHT may provide a good opportunity to raise this sensitive topic.

#### 4.5.10. Non-Compliance 

Non-compliance is frequent in adults with PWS [9] and in those with intellectual disabilities [111]. To improve compliance, it is important to prepare patients for monthly bleedings or spotting, and potential feelings of fear and embarrassment. Additionally, it is important to regularly ask whether the patient is still compliant and to determine reasons for non-compliance. As transdermal patches do not have to be applied daily, these treatment options may lead to better compliance.

#### 4.5.11. Discontinuation of HHT

Most experts in the expert panel discussion recommend to continue HHT until the age of 50 years. Some experts also indicated that HHT could be continued longer or be restarted in case of osteoporosis/osteopenia or when symptoms occurred.

### 4.6. Strengths and Limitations

One strength of our study is the sample size, which is large considering the rarity of the syndrome. Moreover, we provide evidence- and expert-based practical recommendations for the screening and treatment of hypogonadism in PWS, which are urgently needed. A limitation is that many patients already used HHT before their first visit to our outpatient clinic. In these cases, we had to rely on medical files or the memory of the patients or their caregivers to determine whether the patient had hypogonadism. This also led to a large number of missing data for the analysis of laboratory values and the determination of primary or central hypogonadism. Also, we could not assess the effect of hypogonadism on BMD, as we had an insufficient number of DEXA scan results.

## 5. Conclusions

In conclusion, hypogonadism was present in nearly all females with PWS in our cohort (94%). Although adequate treatment is important for wellbeing and particularly bone health [37,38], hypogonadism is often undiagnosed (34%) and untreated. Therefore, we recommend screening for hypogonadism in all women with PWS by assessing the menstrual cycle. If the cycle is irregular or absent, treatment should be considered. To guide the screening and treatment of hypogonadism in women with PWS, we provide a practical algorithm.

## Figures and Tables

**Table 1:** Baseline characteristics of 64 females with Prader-Willi syndrome.

	Hypogonadism Known *n* = 50	Hypogonadism Unknown due to Treatment ^a^ *n* = 10	Hypogonadism Unknown due to Age ^b^ *n* = 4	Total *n* = 64
Age in years, median (IQR)	27 (21–34)	25 (19–38)	56 (51–58)	28 (22–37)
Age range in years	18–52	18–49	49–58	18–58
BMI in kg/m^2^, median (IQR)	32 (27–43)	25 (22–34)	31 (24–34)	32 (27–40)
Genetic subtype				
Deletion	30 (60%)	6 (60%)	1 (25%)	37 (58%)
mUPD ^c^	16 (32%)	3 (30%)	3 (75%)	22 (34%)
ICD	1 (2%)	0 (0%)	0 (0%)	1 (2%)
Unknown	3 (6%)	1 (10%)	0 (0%)	4 (6%)
rhGH treatment ^d^				
Only during childhood	6 (12%)	2 (20%)	0 (0%)	8 (13%)
Only during adulthood	2 (4%)	0 (0%)	0 (0%)	2 (3%)
Both	18 (36%)	6 (60%)	0 (0%)	24 (38%)
Never	24 (48%)	2 (20%)	4 (100%)	30 (47%)
Current rhGH treatment	18 (36%)	6 (60%)	0 (0%)	24 (38%)
Psychotropic medication	16 (32%)	5 (50%)	3 (75%)	24 (38%)
Living situation				
With family	12 (24%)	3 (30%)	0 (0%)	15 (23%)
In a specialized PWS group home	11 (22%)	3 (30%)	1 (25%)	15 (23%)
In a non-specialized facility	27 (54%)	4 (40%)	3 (75%)	34 (53%)
Scholar level				
Secondary vocational education	2 (4%)	2 (20%)	0 (0%)	4 (6%)
Pre-vocational secondary education	1 (2%)	0 (0%)	0 (0%)	1 (2%)
Special education	35 (70%)	6 (60%)	2 (50%)	43 (67%)
No education	2 (4%)	1 (10%)	1 (10%)	4 (6%)
Unknown	10 (20%)	1 (10%)	1 (10%)	12 (19%)
Relationship status				
In a relationship with sexual intercourse	5 (10%)	1 (10%)	0 (0%)	6 (9%)
In a relationship without sexual intercourse	8 (16%)	0 (0%)	1 (25%)	9 (14%)
Not in a relationship	33 (66%)	7 (70%)	2 (50%)	42 (66%)
Unknown	4 (8%)	2 (20%)	1 (25%)	7 (11%)

Abbreviations: body mass index (BMI), paternal deletion (deletion), recombinant human growth hormone (rhGH), imprinting center defect (ICD), interquartile range (IQR), maternal uniparental disomy (mUPD), Prader-Willi syndrome (PWS). Data are presented as *n* (%), unless otherwise specified. **^a^** The presence of hypogonadism could not be assessed as it was unknown whether they had had a regular menstrual cycle before the start of estrogen- and/or progestogen-containing preparations. **^b^** The presence of hypogonadism could not be assessed as these women were already over 50 years old during their first visit to our outpatient clinic and there was no information available about the menstrual cycle before they had reached menopausal age. **^c^** In 6 patients with suspected mUPD, the parents were not available for genetic testing. Therefore, mUPD is the most likely genotype, but an ICD could not be ruled out in these patients. **^d^** Patients older than 25 years old received a starting rhGH dose of 0.3 mg/day, while in patients younger than 25 years old, the starting growth hormone dosage was 1.0 mg/m^2^/day, which was gradually lowered to 0.33 mg/m^2^/day after reaching the final height. However, this dose could be adjusted according to insulin-like growth factor-1 (IGF-1) measurements and clinical effects.

**Table 2:** Hypogonadism in women with Prader-Willi syndrome.

	Women with PWS *n =* 64
Hypogonadism before screening	30/50 (60%)
Of whom untreated number of women with	7/30 (23%)
hypogonadism (%)	
Hypogonadism revealed by screening	17/50 (34%)
Hypogonadism after screening	47/50 (94%)
Of whom untreated number of women with	13/47 (28%)
hypogonadism (%)	
Age at start hypogonadism hormone therapy, median (IQR)	20 (16–28)
	(*n* = 33)
Hypogonadism after screening according to BMI group	
In females with BMI < 25 kg/m^2^	7/7 (100%)
In females with BMI 25–30 kg/m^2^	13/14 (93%)
In females with BMI > 30 kg/m^2^	27/29 (93%)

Abbreviations: body mass index (BMI), interquartile range (IQR), Prader-Willi syndrome (PWS). Data are presented as number of patients with the outcome/number of patients for whom data was available (%), unless otherwise specified.

**Table 3:** LH, FSH and estradiol values in 27 women with Prader-Willi syndrome and hypogonadism.

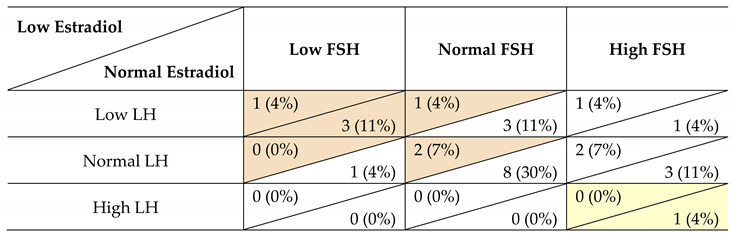

Abbreviations: follicle stimulating hormone (FSH), luteinizing hormone (LH). Data are presented as *n* (%). Laboratory measurements were available for 27 women with hypogonadism. In the other females not all three laboratory measurements were available, only laboratory measurements during the use of estrogen- and/or progestogen-containing preparations were available or the patient was over 50 years old. The type of hypogonadism is indicated with colors, where orange represents central hypogonadism and yellow represents primary hypogonadism. In the other patients, the hypogonadism could not be classified as either central or primary due to discrepant LH and FSH values. ‘Low’ refers to laboratory values below reference range. ‘Normal’ refers to laboratory values within the reference range (which may be inadequately low in case of low estradiol levels). ‘High’ refers to laboratory concentrations above the reference range.

**Table 4:** Literature review of hypogonadism in women with Prader-Willi syndrome (Part 1).

Article	*n*	Country	Age Range (years)	Genotype (Deletion/mUPD/ICD/ Translocation)	Mean BMI (kg/m^2^)	Assays Used	Definition Hypogonadism
Partsch et al. (2000) [13]	9 ^a^	Germany	18–34 ^b^	NA/NA/0/0	46 ^c^	Commercially available immunoassays	Low estradiol levels and absence of a regular MC
Whittington et al. (2002) [14] ^d^	24	United Kingdom	18–47	NA ^e^	NA	NA	absence of a regular MC
Grugni et al. (2003) [15]	20	Italy	18–28	13/7/0/0	44	LH/FSH: immunochemiluminescent assays Estradiol: chemiluminescent immunoassay	absence of a regular MC
Höybye et al. (2005) [16] ^d^	6	Sweden	19–37	NA ^e^	Median 36	Commercially available immunoassays	low estradiol, absence of a spontaneous regular MC, or treatment with sex steroids
Miller et al. (2008) [17] ^d^	6	Florida, USA	18–29	4/2/0/0	32	Commercially available radioimmunoassays	Hypogonadotropic hypogonadism: delayed onset of puberty (>13 year) in addition to low gonadotropin levels for age
Brandau et al. (2008) [18] ^d^	21	Missouri, USA	18–50	14/7/0/0	33	FSH, LH: chemiluminescence assays Estradiol: radioimmunoassay	low estradiol levels
Sode-Carlsen et al. (2010) [19] ^d^	24	Denmark, Norway, Sweden	18–41	9/2/1/0 (12 NA) ^e^	Median 28	Commercially available immunoassays	low estradiol, absence of a spontaneous regular MC, or treatment with sex steroids
Van Nieuwpoort et al. (2011) [20]	11	The Netherlands	19–41	14/1/0/0 ^c^	33	Commercially available immunoassays	absence of a spontaneous regular MC
Hirsch et al. (2015) [21] ^d^	19	Israel	18–47	10/8/1/0	33	LH, FSH, testosterone, estradiol: immunoassays Inhibin B, AMH: ELISA SHBG: immunochemiluminescence	absence of a regular MC
Coupaye et al. (2016) [22] ^d^	35	France	18–58 ^c^	42/24/0/0 ^c,f^	39 ^c^	Routine techniques	absence of a spontaneous regular MC, treatment with sex steroid or estradiol level < 120 ng/L at any time

Abbreviations: anti-Müllerian hormone (AMH), body mass index (BMI), paternal deletion (deletion), two-site enzyme-linked immunosorbent assays (ELISA), follicle stimulating hormone (FSH), imprinting center defect (ICD), luteinizing hormone (LH), menstrual cycle (MC), maternal uniparental disomy (mUPD), not available (NA), sex hormone-binding globulin (SHBG), United States of America (USA). Only data on adult women with PWS are reported. The authors were contacted if separate data on only adults was not presented in the article. ^a^ Twelve women were included in this study, but in three women hypogonadism could not be investigated as they already received sex hormone replacement therapy. ^b^ Age range for all twelve women included in the study. ^c^ Data for all males and females included in this study. ^d^ Additional data was provided by the authors. ^e^ All methylation-positive. ^f^ Only patients a deletion or an mUPD were included.

**Table 5:** Literature review of hypogonadism in women with Prader-Willi syndrome (Part 2).

Article	Hypogonadism *n* (%)	Primary/ Central Hypogonadism	FSH	LH	Estradiol	SHBG	Inhibin B	AMH
Partsch et al. (2000) [13]	9 (100%)	- ^a^	-	-	-	-	-	-
Whittington et al. (2002) [14]	20 (100%) (4 NA)	-	-	-	-	-	-	-
Grugni et al. (2003) [15]	17 (85%)	-	2.1 (0.1–5.1) IU/L	1.3 (0.1–5.0) IU/L	34 (15–72) pg/mL *123 (55–264) pmol/L*	-	-	-
Höybye et al. (2005) [16]	5 (83%)	0/5	4.9 (1.0–7.8) IU/L	2.1 (0.6–5.5) IU/L	104 (72–203) pmol/L	-	-	-
Miller et al. (2008) [17]	6 (100%)	1/5	-	-	-	-	-	-
Brandau et al. (2008) [18]	14 (70%) (1 NA)	-	3.8 (0.4–15.0) IU/L	1.8 (0.1–5.3) IU/L	23 (5–82) pg/mL *85 (18–301) pmol/L*	-	-	-
Sode-Carlsen et al. (2010) [19]	13 (54%)	1/5 (7 NA)	4.9 (<0.2–17.6) IU/L	2.7 (<1.0–12.9) IU/L	0.13 (0.08–0.54) nmol/L *130 (80–54) pmol/L*	-	-	-
Van Nieuwpoort et al. (2011) [20]	9 (81%)	0/4 (5 NA)	Median (IQR) 4.65 (3.49) IU/L	Median (IQR) 2.75 (2.26) IU/L	Median (IQR) 92 (257) pmol/L	Median (IQR) 25.5 (20.2) nmol/L	-	-
Hirsch et al. (2015) [21]	18 (95%)	1/2 (15 combined ^b^)	6.1 (0.5–18.3) IU/L	2.6 (0.1–6.8) IU/L	144 (37–733) pmol/L	47.1 (5.1–146.0) nmol/L	26.9 (10.0–73.0) pg/mL (*n* = 18)	1.04 (0.02–2.75) ng/mL (*n* = 17)
Coupaye et al. (2016) [22]	33 (94%)	-	Mean ± SD 6.4 ± 9.6 IU/L	Mean ± SD 4.2 ± 4.3 IU/L	50 (12–143) ng/L *183 (44–525) pmol/L*	Mean ± SD 36.9 ± 26.4 nmol/L	Mean ± SD 5.6 ± 6.0 pg/mL (*n* = 5)	Mean ± SD 0.9 ± 0.6 ng/mL (*n* = 5)

Abbreviations: anti-Müllerian hormone (AMH), follicle stimulating hormone (FSH), luteinizing hormone (LH), International System of Units (SI), interquartile range (IQR), not available (NA), standard deviation (SD), sex hormone-binding globulin (SHBG). When laboratory measurements were reported in non-SI units, the converted values are shown in *italics*. Only values for FSH, LH, and estradiol in patients that did not use estrogen- and/or progestogen-containing preparations during blood withdrawal are included. All laboratory values are presented as mean (range), unless otherwise specified. Values that were below the detection limit were considered equal to the detection limit to calculate the mean. ^a^ Gonadotropin levels were subnormal in all but one patient (of the total population of 7 males and 12 females) and showed a reduced responsiveness to stimulation with exogenous gonadotropin-releasing hormone. ^b^ Combined primary and central hypogonadism.

## Data Availability

The datasets generated during and/or analyzed during the current study are not publicly available but are available from the corresponding author on reasonable request.

## Supplementary Materials

The following are available online at https://www.mdpi.com/article/10.3390/jcm10245781/s1, Table S1: Full search strategy (Embase), Table S2: Laboratory values in women with Prader-Willi syndrome according to genotype and GH treatment, Table S3: Laboratory values in women with Prader-Willi syndrome according to BMI and age, Figure S1: LH, FSH and estradiol values for 27 women with Prader-Willi syndrome and hypogonadism.
