# Supplementary material for: Hypogonadism in Women with Prader-Willi Syndrome—Clinical Recommendations Based on a Dutch Cohort Study, Review of the Literature and an International Expert Panel Discussion
Source: J Clin Med. 2021 Dec 10;10(24):5781. doi: 10.3390/jcm10245781 (PMC8707541; doi:10.3390/jcm10245781)
Supplement: Supplementary file 1 [file jcm-10-05781-s001.zip › jcm-1463289-supplementary.pdf]

Supplementary Materials

Table S1. Full search strategy (Embase)

('Prader Willi syndrome'/exp OR 'mkrn3 gene'/de OR 'makorin ring finger protein 3'/de OR 'magel2 gene'/de OR 'magel2 protein'/de OR 'necdin'/de OR 'small nuclear ribonucleoprotein polypeptide n'/de OR 'snrpn gene'/de OR 'snrpn protein'/de OR 'e6 associated protein'/de OR ((Prader* NEAR/3 Willi*) OR PraderWilli OR mkrn3 OR makorin-3 OR znf127 OR D15S9 OR RNF63 OR EC-2.3.2.27 OR ZFP127 OR CPPB2 OR magel2 OR magel-2 OR ((makorin) NEAR/3 (ring) NEAR/3 (3)) OR ((zinc) NEAR/3 (finger) NEAR/3 (127)) OR ((ring) NEAR/3 (finger) NEAR/3 (63)) OR ((MAGE OR melanoma) NEAR/3 (family) NEAR/3 (L2)) OR ((necdin-like OR MAGE-like) NEAR/3 (protein) NEAR/3 (1 OR 2)) OR NDNL1 OR MAGE-Like-2 OR SHFYNG OR PWLS OR NM15 OR (NDN NOT nonclassic-differentiation-number*) OR necdin* OR HsT16328 OR PWCR OR C15orf2 OR NPAP1 OR NPAP-1 OR Nuclear-Pore-Associated-Protein-1 OR ((chromosome-15) NEAR/3 (open-reading-frame* OR ORF*) NEAR/3 (2)) OR SNURF-SNRPN* OR SNRPN* OR Small-Nuclear-Ribonucleoprotein-Polypeptide-N* OR Small-Nuclear-Ribonucleoprotein-Associated-Protein-N* OR Tissue-Specific-Splicing-Protein* OR Sm-Protein-D OR SM-Protein-N OR HCERN3 OR (((Sm-N OR SMN OR Sm-D OR PET1 OR BEY OR PED) AND (gene OR genes OR genetic OR genome* OR genomic)) NOT (sec*-malign*-neoplas* OR survival-motor-neuro*)) OR Small-Nucl*-Ribonucleoprot*-N OR RT-LI OR PWCR OR SNORD107 OR SNORD-107 OR (("C/D" OR CD) NEAR/3 (box*) NEAR/3 (107 OR 64 OR 109A OR 116 OR 115 OR 109B)) OR HBII-436 OR RF01164 OR SNORD64 OR HBII-13 OR RF00570 OR SNORD109A OR HBII-438A OR RF01278 OR SNORD116 OR HBII-85 OR PWCR1 OR SNORD115 OR HBII-52 OR RNHBII52 OR RF00105 OR SNORD109B OR HBII-438B OR RF01278 OR KIAA1899):ab,ti,kw) AND ('gonadal disease'/exp OR 'steroid receptor'/exp OR 'bone characteristics and functions'/exp OR 'sex hormone binding globulin'/de OR 'luteinizing hormone derivative'/exp OR 'follitropin'/exp OR 'inhibin'/exp OR 'infertility'/exp OR 'sexual dysfunction'/exp OR 'sex'/exp OR 'gonadorelin derivative'/exp OR 'gonadorelin receptor'/de OR 'luteinizing hormone receptor'/de OR 'menstruation disorder'/exp OR 'menstrual cycle'/exp OR 'birth control'/de OR 'contraception'/exp OR 'safe sex'/exp OR 'contraceptive device'/exp OR 'genital system'/exp OR 'steroid'/exp OR 'Muellerian inhibiting factor'/de OR 'menopause and climacterium'/exp OR 'ovary polycystic disease'/de OR 'wolffian duct'/de OR 'sexual characteristics'/exp OR 'breast'/exp OR 'breast development'/de OR 'endocrine breast disease'/exp OR 'breast malformation'/exp OR 'hair'/exp OR 'genital system function'/exp OR 'pregnant woman'/de OR 'reproduction'/de OR 'sexual dysfunction'/exp OR 'osteoporosis'/exp OR 'osteopenia'/exp OR 'bone characteristics and functions'/exp OR 'fracture'/exp OR 'aggression'/exp OR 'dual energy X ray absorptiometry'/de OR 'problem behavior'/de OR 'behavior disorder'/de OR 'hypersexuality'/exp OR 'genital system disease'/exp OR 'genital malformation'/exp OR 'orchidopexy'/de OR 'spontaneous abortion'/de OR 'prolactin derivative'/exp OR 'precocious puberty'/exp OR 'alpha fetoprotein'/de OR 'bone maturation'/de OR 'aromatization'/de OR 'aromatase deficiency'/de OR 'aromatase'/de OR 'cholesterol monooxygenase (side chain cleaving)'/de OR 'steroid 17alpha monooxygenase'/de OR '3(or 17)beta hydroxysteroid dehydrogenase'/de OR (gonad* OR hypogonad* OR hypogenital* OR hypergonad* OR eugonad* OR normogonad* OR hypergenital* OR androgen* OR estrone OR estriol OR trihydroxyestrin* OR estrogen* OR estradiol* OR androst* OR dihydrotestosteron* OR dehydroepiandrosteron* OR prasteron* OR dehydroisoandrosteron* OR testosteron* OR hydroxypregnenolone* OR pregnenolone* OR progesteron* OR progestin* OR SHBG OR sex-hormone-binding-globulin* OR LH OR luteinizing-hormone* OR luteinising-hormone* OR lutropin* OR FSH OR follitropin* OR follicle-stimulating-hormone* OR inhibin* OR ((pregnan*) NEAR/3 (woman OR women) NEAR/3 (with-IDD OR with-ID OR with-Prader)) OR fertil* OR infertil* OR infecund* OR sex OR sexual* OR orgasm* OR transsexual* OR bisexual* OR heterosexual* OR homosexual* OR gay OR straight OR lesbian* OR GnRH OR choriogonadotropin-receptor* OR amenorrhea* OR dysmenorrhea* OR menorrhagia* OR metrorrhagia* OR oligomenorrhea* OR menstrual* OR premenstrual* OR menstruat* OR contracept* OR condom* OR birth-control* OR IUD OR intrauterine-device* OR depo-provera* OR ovulat* OR ovar* OR testis OR testes OR testicular* OR ((granulosa* OR follicular* OR theca* OR sertoli* OR Leydig* OR luteal* OR cumu-

lus*) NEAR/3 (cell*)) OR follic* OR phallic* OR Müllerian* OR Muellerian* OR fallopian* OR cervix OR endocervix OR cervical OR uterus OR uterine OR vagina* OR penis* OR penile OR phallus OR prostate* OR scrotum* OR sperm* OR steroid* OR menopaus* OR premenopaus* OR perimenopaus* OR postmenopause* OR climacterium* OR galactorrhea* OR PCOS OR ((polycystic*) NEAR/3 (ovar*) NEAR/3 (syndrome* OR disease*)) OR ((Stein) NEXT/3 (Leventhal)) OR ((wolff* OR archinephric* OR mesoneph*) NEAR/3 (duct*)) OR puberty OR pseudopuberty OR pubescence OR genital* OR breast* OR mamma* OR nipple* OR thelarche* OR hair* OR beard* OR gynecomast* OR gynaecomast* OR ejaculat* OR erection* OR erectile* OR sperm* OR libido* OR orgasm* OR anorgasm* OR osteoporo* OR ((bone*) NEAR/3 (densit* OR mass* OR health* OR strength* OR matur*)) OR fractur* OR aggress* OR DEXA OR DXA OR ((dual-energy) NEAR/3 (x-ray OR xray OR roentgen-ray)) OR ((problem* OR challeng*) NEAR/3 (behavior* OR behaviour*)) OR hypersexual* OR hyposexual* OR micropenis* OR orchidopex* OR orchiopex* OR ((luteal* OR secretor* OR premenstrual* OR proliferative*) NEXT/1 (phase*)) OR endomet* OR corpus-luteum* OR menarche* OR miscarriage* OR spontane*-abortion* OR prolactin* OR semiferous* OR andropause* OR virilization* OR androstane* OR phallic* OR ((seminal* OR semen*) NEAR/3 (vesic*)) OR ((male* OR man OR men OR boy*) NEAR/3 (feminization* OR feminisation*)) OR ((female* OR woman OR women OR girl*) NEAR/3 (masculinization* OR masculinisation*)) OR alpha-fetoprotein* OR pubarche* OR clitoris* OR 5-alpha-reductase* OR 5alpha-reductase* OR aromat* OR androstanedio* OR ((cholesterol*) NEAR/3 (side-chain-cleav* OR monooxygenase* OR desmolase*)) OR CYP11A1 OR P450scc OR ((17α OR 17 OR 17alpha) NEAR/3 (hydroxylase* OR monooxygenase* OR desmolase*)) OR 17-20-lyase OR ((3β OR 17β OR 3beta OR 17beta OR 3-beta OR 17-beta) NEXT/1 (hydroxysteroid) NEXT/3 (dehydrogenase* OR oxidoreductase*)) OR 3β-HSD OR 17β-HSD OR 3beta-HSD OR 17beta-HSD OR 3-beta-HSD OR 17-beta-HSD OR reproduction* OR reproductive*):ab,ti,kw) OR (('Prader Willi syndrome'/exp OR ((Prader* NEAR/3 Willi*) OR PraderWilli):ab,ti,kw) AND ('BRCA1 protein'/de OR 'BRCA2 protein'/de OR (BRCA1 OR BRCA2):ab,ti,kw)) NOT ([Conference Abstract]/lim AND [1800-2017]/py) AND [English]/lim

Similar search terms with an adjusted search strategy were used for the databases Medline (Ovid), Web of Science Core Collection, and the Cochrane Central Register of Controlled Trials. Additionally, references were searched. We identified articles describing hypogonadism in both males and females with PWS. For this manuscript, we manually excluded articles that only described hypogonadism in males with PWS.

Table S2. Laboratory values in women with Prader-Willi syndrome according to genotype and GH treatment

	Number of Observations	Total *n* = 64	Deletion *n* = 37	mUPD *n* = 24	*p*-Value	Current GH Treatment *n* = 24	No current GH Treatment *n* = 40	*p*-Value	*p*-Value Corrected for Age
Estradiol (pmol/L)	34	71 (38–121)	69 (36–177)	87 (58–121)	0.7	64 (26–128)	82 (41–118)	0.4	0.3
LH (IU/L)									
Before 01-02-2019	27	2.4 (1.0–5.4)	2.9 (1.5–5.3)	1.5 (0.3–7.8)	0.9	3.6 (2.3–7.1)	1.6 (0.5–5.3)	0.4	0.2
After 01-02-2019	6	2.6 (1.2–5.4)	1.8 (0.5–9.6)	3.2 [a]		3.0 [a]	2.1 (0.8–7.7)		
FSH (IU/L)									
Before 01-02-2019	26	6.4 (3.3–8.5)	5.3 (3.0–7.5)	6.3 (3.6–9.8)	0.9	7.5 (7.0–10.0)	5.0 (2.9–8.2)	0.4	0.2
After 01-02-2019	8	5.5 (0.2–8.5)	6.7 (1.2–21.0)	0.1, 0.6, 7.5 [a]		6.4 [a]	4.6 (0.1–8.8)		
SHBG (nmol/L)	47	53 (30–73)	54 (30–81)	45 (27–60)	0.4	54 (42–68)	51 (23–75)	0.5	0.3

Abbreviations: paternal deletion (deletion), follicle stimulating hormone (FSH), growth hormone (GH), luteinizing hormone (LH), maternal uniparental disomy (mUPD), sex hormone-binding globulin (SHBG). Data are presented as median (interquartile range). Laboratory values for LH, FSH, and estradiol are given for patients at baseline or during the last measurement available before the start of estrogen- and/or progestogen-containing preparations. For SHBG the measurement closest to baseline is given. Values that were below the measuring threshold were considered equal to the measuring threshold to calculate the mean. For example, when FSH was below 0.5, this was considered 0.5. *P*-values are calculated with age and BMI as continuous variables. [a] Individual measurements are given as there were too few observations to calculate a median and IQR.

Table S3. Laboratory values in women with Prader-Willi syndrome according to BMI and age

	BMI < 25 kg/m² *n* = 13	BMI 25–30 kg/m² *n* = 16	BMI > 30 kg/m² *n* = 35	*p*-Value	*p*-Value Corrected for Age	Age < 25 years *n* = 26	Age 25–30 years *n* = 13	Age > 30 years *n* = 25	*p*-Value
Estradiol (pmol/L)	50 (23–129)	78 (22–108)	78 (52–144)	0.2	0.4	69 (48–131)	86 (36–381)	73 (28–126)	0.4
LH (IU/L)									
Before 01-02-2019	2.4, 4.2, 8.7 [a]	2.9 (1.4–6.4)	1.6 (0.5–5.3)	0.04	0.051	2.9 (0.1–4.6)	2.4 (1.5–5.8)	1.6 (0.8–6.4)	0.3
After 01-02-2019	3.0 [a]	NA	2.1 (0.8–7.7)			1.5, 3.0 [a]	NA	2.7 (0.6–9.9)	
FSH (IU/L)									
Before 01-02-2019	7.5, 11.1 [a]	7.5 (5.3–8.8)	4.4 (3.0–7.3)	0.08	0.06	7.5 (4.4–8.8)	7.5 (2.5–8.6)	5.4 (3.2–8.2)	0.2
After 01-02-2019	6.4 [a]	0.6 [a]	6.1 (0.1–12.9)			0.1, 4.6, 6.4 [a]	NA	7.5 (0.3–17.0)	
SHBG (nmol/L)	63 (56–84)	59 (26–89)	38 (23–59)	0.01	0.08	53 (29–85)	47 (19–77)	53 (33–68)	0.9

Abbreviations: body mass index (BMI), follicle stimulating hormone (FSH), luteinizing hormone (LH), not available (NA), sex hormone-binding globulin (SHBG). Data are presented as median (IQR). Laboratory values for LH, FSH, and estradiol are given for patients at baseline or during the last measurement available before the start of estrogen- and/or progestogen-containing preparations. For SHBG the measurement closest to baseline is given. Values that were below the measuring threshold were considered equal to the measuring threshold to calculate the mean. For example, when FSH was below 0.5, this was considered 0.5. *P*-values are calculated with age and BMI as continuous variables. [a] Individual measurements are given as there were too few observations to calculate a median and IQR.

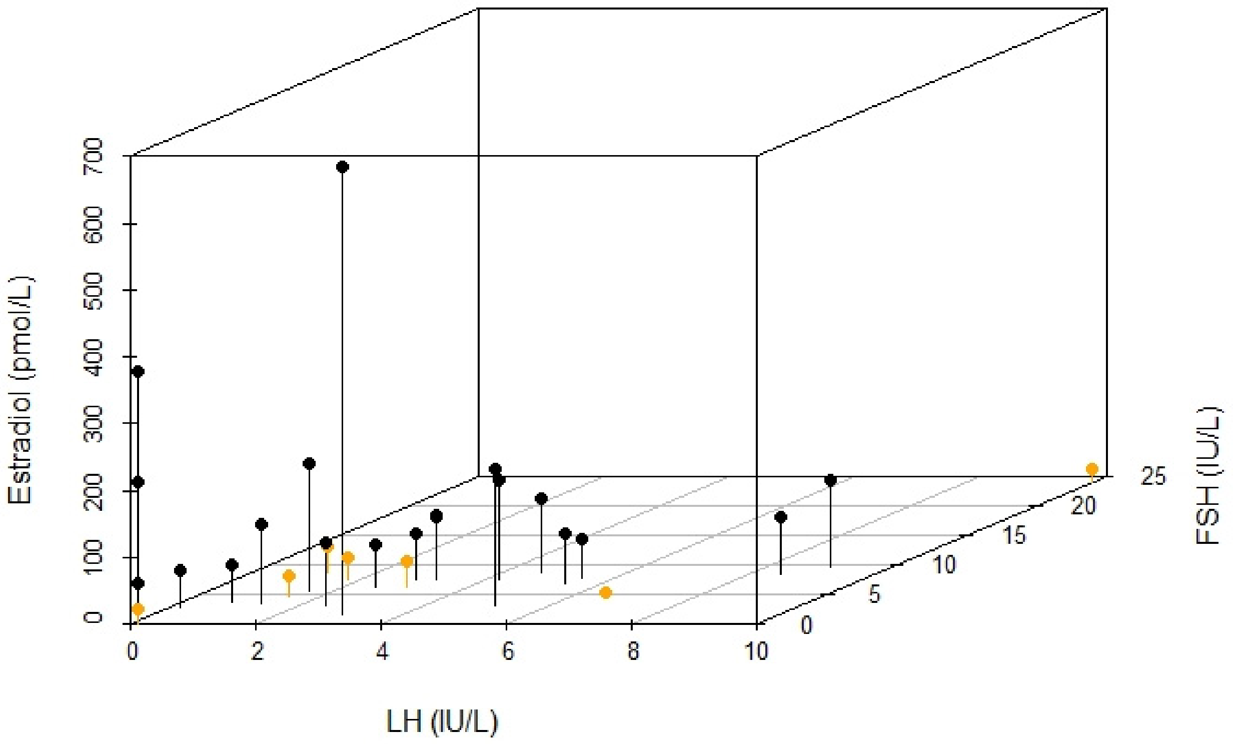

Figure S1. LH, FSH and estradiol values for 27 women with Prader-Willi syndrome and hypogonadism.
Abbreviations: follicle stimulating hormone (FSH), luteinizing hormone (LH). In orange are women with an estradiol value below the reference range and in black the women with an estradiol value above the reference range.
